# Intermittent hypoxia interventions for inducing ventilatory acclimatization to high altitude?

**DOI:** 10.1113/EP093689

**Published:** 2026-10-11

**Authors:** Martin Burtscher, Axel Kleinsasser, Johannes Burtscher, Katharina Hüfner, Erik R. Swenson, Peter Hackett, Hannes Gatterer

**Affiliations:** ^1^ Department of Sport Science University of Innsbruck Innsbruck Austria; ^2^ Department of Anesthesiology & Critical Care Medicine Innsbruck Medical University Innsbruck Austria; ^3^ Department of Psychiatry Psychotherapy, Psychosomatics and Medical Psychology University Hospital for Psychiatry II Medical University of Innsbruck Innsbruck Austria; ^4^ Department of Medicine Geisel School of Medicine at Dartmouth Lebanon New Hampshire USA; ^5^ Department of Medicine University of Colorado Anschutz Medical Campus Aurora Colorado USA; ^6^ Institute of Mountain Emergency Medicine Eurac Research Bolzano Italy

**Keywords:** acclimatization, altitude, hypoxia, intermittent, renal compensation, ventilatory response

## Abstract

Accumulation of ∼200 h of hypoxia exposure (pre‐acclimatization) has been suggested to prevent the development of acute mountain sickness when a person is subsequently exposed to high altitude. These pre‐exposures are typically undertaken intermittently at or near the place of residence, primarily during sleep in hypoxic tents, and are likely to induce effective ventilatory acclimatization. However, the physiological consequences of different modes of intermittent hypoxia (IH) exposure on ventilatory acclimatization remain largely obscure and are the subject of much controversy. The aim of this review is therefore to summarize and discuss the findings of relevant studies and draw on our long‐term practical experience to address the effects of IH exposure on ventilatory acclimatization and to provide suggestions for effective pre‐acclimatization. We provide a balanced overview of studies investigating the effects of different types of altitude/hypoxia exposure on the hypoxic ventilatory response and renal compensation to hyperventilation‐induced respiratory alkalosis. Although it has been demonstrated convincingly that the hypoxic ventilatory response can increase by 50%–100% with only a few short IH sessions (1 h per day for 7 days), the available data on the effects of IH on renal compensation do not permit definitive conclusions to be drawn. Long‐term exposure to IH over several weeks appears to induce renal compensation effectively, allowing for a full ventilatory response and favourable oxygenation at high altitude. To clarify definitively which strategies are most suitable, long‐term systematic investigations of various IH strategies are urgently needed.

## Abstract (highlights)

- **What is the topic of this review?**
  This review provides a summary of the findings of relevant studies on the effects of continuous and, in particular, intermittent hypoxia exposure on ventilatory acclimatization.
- **What advances does it highlight?**
  The hypoxic ventilatory response can increase by 50%–100% with only a few short intermittent hypoxia sessions (e.g., 1 h per day for 7 days), whereas long‐term exposure to intermittent hypoxia over several weeks appears to induce renal compensation effectively, allowing for a full ventilatory response and favourable oxygenation at high altitude.

## 1 BACKGROUND

Exposure to an environment with reduced oxygen, such as high altitudes, provokes increased pulmonary ventilation (${\dot{V}}_{E}$), representing a rapid defensive response against impending tissue hypoxia (Oeung et al., 2023; Sato et al., 1992; Weil et al., 1970). Therefore, inducing ventilatory acclimatization might be beneficial in preparing for treks or high‐altitude expeditions. Ventilatory acclimatization is characterized by a progressive increase in pulmonary ventilation during sustained or repeated exposure to hypoxia, driven by enhanced hypoxic ventilatory sensitivity and facilitated by renal bicarbonate loss, which compensates for respiratory alkalosis. We recently suggested that spending ∼200 h in a hypoxic, simulated‐altitude environment prior to a trip might substantially reduce the risk of acute mountain sickness (AMS) upon subsequent exposure to terrestrial high altitude (J. Burtscher et al., 2026), although this analysis was based on extrapolated results and requires experimental verification. Hypoxic exposure is typically conducted intermittently, for example during sleep in hypoxic tents, with the level of hypoxia increasing progressively (J. Burtscher et al., 2026; Tannheimer & Lechner, 2020). Ventilatory acclimatization is completed within the first 6–10 days at high altitude (continuous hypoxia), and this time frame aligns well with the 200 h accumulated intermittently (J. Burtscher et al., 2026; Lenfant & Sullivan, 1971; Mallet et al., 2023; Sato et al., 1994). Therefore, it is reasonable to suggest that spending this amount of time in a hypoxic environment is adequate to achieve ventilatory acclimatization and, consequently, tolerance to hypoxia. Ventilatory acclimatization is characterized by an augmentation in the ventilatory response to hypoxia (HVR) (Powell et al., 2000). The hypoxia‐inducible factor‐2α protein plays a key role in the gain of HVR, which is attributed to adaptive functional and molecular changes in the carotid bodies and central respiratory pathways that increase their responsiveness to hypoxia (Powell & Fu, 2008). However, hypoxia‐inducible factor‐2α responses and the associated increase in HVR depend on the type of hypoxic exposure, such as continuous versus intermittent or short versus long term (Bishop & Ratcliffe, 2025; Powell & Fu, 2008). Initially, the HVR effect on ventilation is limited by central chemoreceptors in response to a decrease in the partial pressure of arterial carbon dioxide ($P_{\mathrm{aC}O_{2}}$). It is only through renal bicarbonate excretion (renal compensation) that full ventilatory acclimatization can gradually develop, thereby increasing blood oxygenation. It is not yet clear which characteristics of intermittent hypoxia (IH) exposures, such as the duration and intensity of cycles, sleep and wakefulness or rest and exercise, would have the greatest impact on HVR and renal compensation and the associated time course of ventilatory acclimatization. Therefore, the primary objective of this review is to summarize the current evidence on the effects of IH interventions on ventilatory acclimatization in preparation for high‐altitude exposure. For comparative purposes, we also provide an overview of the effects of continuous high‐altitude exposure on HVR and renal compensation, topics that have been investigated in the literature more extensively.

## 2 TWO CASE STUDIES AS CATALYSTS

This brief review was prompted by observations made in our laboratory, illustrated by the two case studies below.

### 2.1 Short‐term pre‐acclimatization (Case 1)

An enthusiastic female mountaineer with a history of repeated high‐altitude pulmonary oedema (HAPE) above 2000 m planned to trek in the Himalayas again and visited our laboratory. We exposed her to a simulated altitude of about 2500–4000 m (inspiratory oxygen fraction ($F_{IO_{2}}$) between 15% and 12%) for 30–60 min on five occasions, during which we observed a marked increase in peripheral oxygen saturation ($S_{pO_{2}}$) from session 1 to session 5 (Figure 1). Subsequently, while under medical observation, she travelled to the Himalayas and tolerated altitudes of ≤5000 m without any signs of HAPE (M. Burtscher et al., 2008). This observation shows that individuals who are particularly sensitive and generally have a low HVR, for example, clearly below the normal mean of 0.98 L/min/$S_{pO_{2}}$ (Oeung et al., 2023), can experience a significant increase in their HVR, and thus their tolerance of high‐altitude hypoxia, after only a few hypoxic exposures.

**FIGURE 1:**
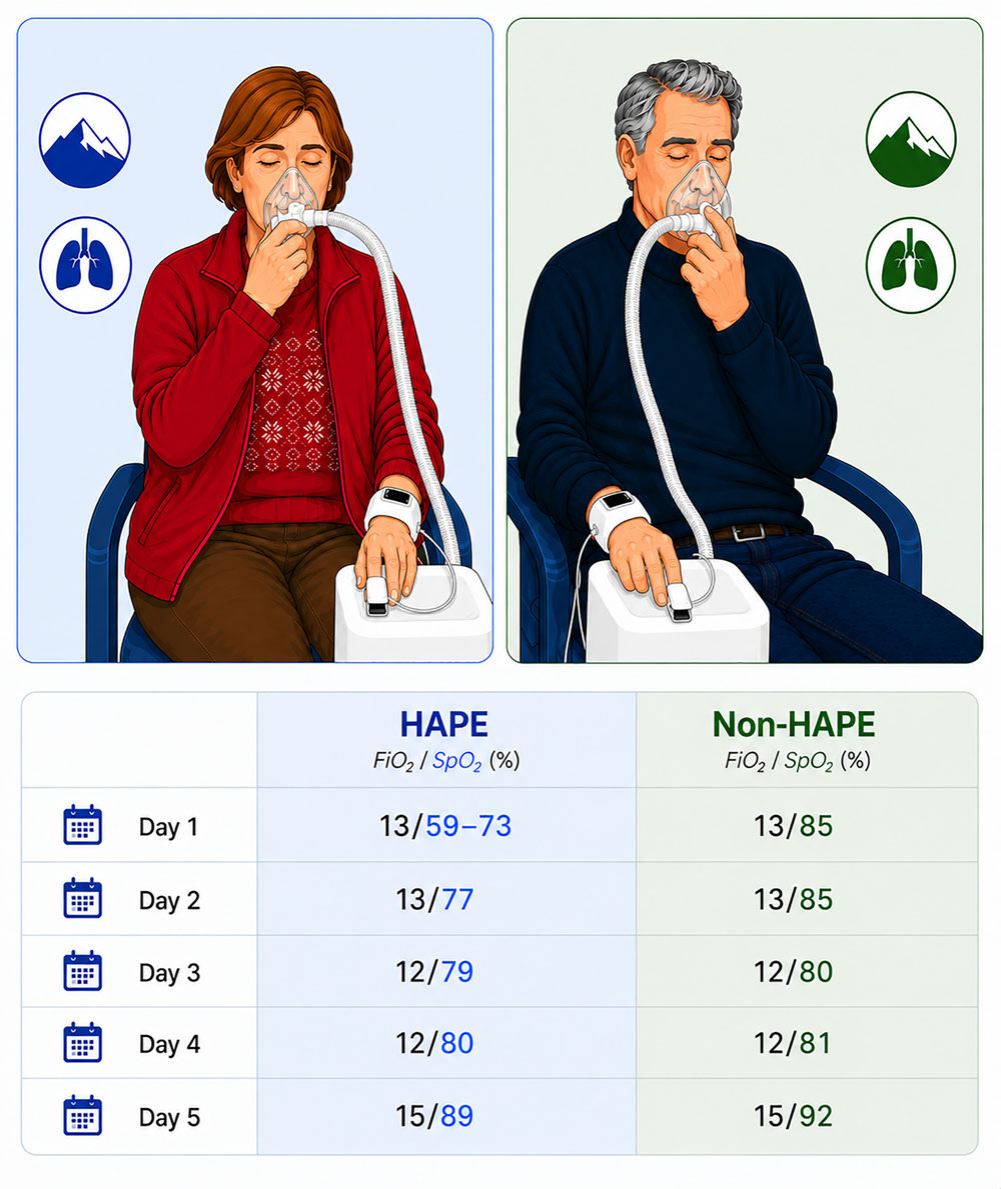
Responses of $S_{pO_{2}}$ to daily 0.5–1.0 h hypoxia exposure for 5 days in a HAPE‐susceptible individual and a non‐susceptible individual. Abbreviations: $F_{IO_{2}}$, inspiratory fraction of oxygen; HAPE, high‐altitude pulmonary oedema; $S_{pO_{2}}$, peripheral oxygen saturation.

### 2.2 Long‐term pre‐acclimatization (Case 2)

Another case study involving long‐term normobaric hypoxia conditioning has prompted many mountaineers to acclimatize in a hypoxic tent prior to embarking on a high‐altitude expedition (M. Burtscher et al., 2022). Prior to his planned Everest expedition, the mountaineer spent 60 nights, each lasting ∼7 h, in a hypoxic tent. Figure 2 shows the hypoxia levels and resting $S_{pO_{2}}$ responses measured in the morning in the hypoxic tent. Based on the recorded $S_{pO_{2}}$ values, it appears that a decay of the initial ventilatory acclimatization occurred during sleep after a few days at an $F_{IO_{2}}$ of 14% (∼3000 m). This statement is based on the assumption that changes in $S_{pO_{2}}$ are primarily the result of changes in ventilation. Further reduction of the $F_{IO_{2}}$ to 11% (∼5300 m) did not result in a further decrease in $S_{pO_{2}}$, but the climber felt unwell. After 25 days, increasing the $F_{IO_{2}}$ to 14% significantly improved $S_{pO_{2}}$, and subsequently reducing it to 11% did not decrease $S_{pO_{2}}$. Given that we have made similar observations in other climbers, we suggested that this multi‐week, intermittent exposure to hypoxia is necessary for proper acclimatization to the altitude of Everest Base Camp. This pre‐acclimatization strategy (in a modified form) has been applied successfully to numerous Everest Flash expeditions involving >300 expeditioners, with no cases of AMS observed (personal communication, 2026) (J. Burtscher et al., 2026). A recent review of the literature suggests that 200 h of hypoxia exposure is likely to be sufficient to largely prevent the development of AMS, at least up to ∼6000 m (J. Burtscher et al., 2026).

**FIGURE 2:**
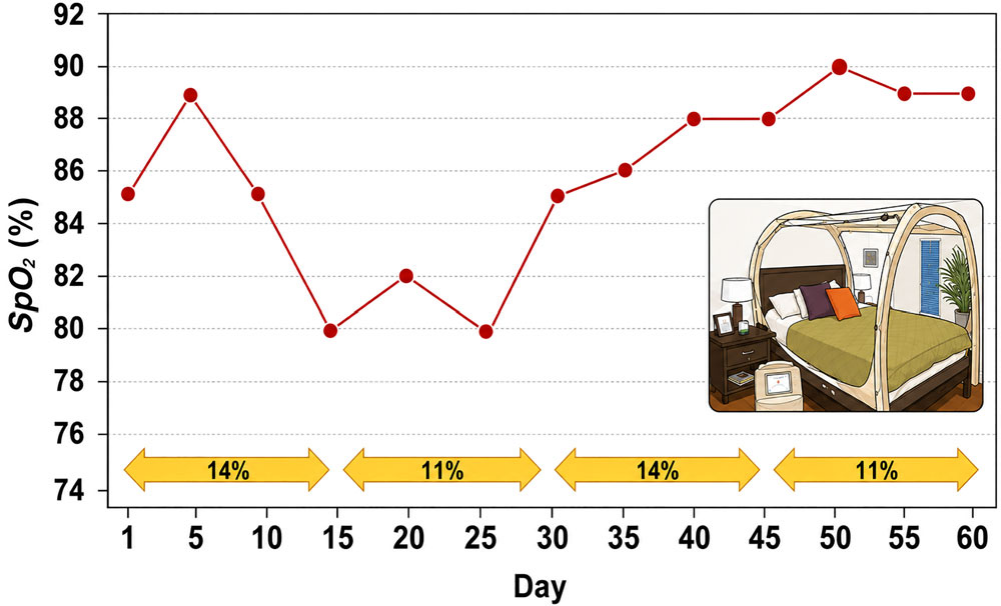
Responses of $S_{pO_{2}}$ to 60 nights sleeping in a hypoxic tent with different levels of $F_{IO_{2}}$. Abbreviations: $F_{IO_{2}}$, inspiratory fraction of oxygen; $S_{pO_{2}}$, peripheral oxygen saturation.

In the first case, a HAPE‐susceptible person clearly benefitted from a short‐term intervention by increasing HVR, whereas the situation in the second case is more complex. Long‐term intervention with nocturnal hypoxia exposures resulted in a significant improvement in $S_{pO_{2}}$ levels at simulated high altitudes only after several weeks. In contrast to the first case, other factors besides an increase in HVR might have contributed to ventilatory acclimatization, including renal compensation for respiratory alkalosis and/or changes in the hypercapnic ventilatory response. The question arises of which individuals would benefit from a short‐term acclimatization process (e.g., involving an increase in HVR) and which individuals would require a more protracted acclimatization process (i.e., complete ventilatory acclimatization).

## 3 GENERAL ASPECTS OF VENTILATORY ACCLIMATIZATION

In normoxic conditions [arterial O_2_ partial pressure ($P_{aO_{2}}$) > 60 mmHg], ventilatory control is predominantly regulated by changes in arterial carbon dioxide tension ($P_{\mathrm{aC}O_{2}}$). However, when $P_{aO_{2}}$ decreases to ∼60 mmHg or below, the ventilatory response becomes substantially more pronounced owing to activation of the HVR, and hypoxaemia becomes the dominant stimulus, overriding the influence of $P_{\mathrm{aC}O_{2}}$ (Oeung et al., 2023; Risdall & Gradwell, 2008). Low arterial $P_{aO_{2}}$ levels are detected by peripheral chemoreceptors (carotid bodies), triggering afferent input to the respiratory control centres. The resulting reflexive activation of the respiratory muscles causes increases in both the volume of air inhaled (tidal volume) and the breathing rate (Pamenter & Powell, 2016). The relationship between $P_{aO_{2}}$ and the arterial or peripheral O_2_ saturation ($S_{aO_{2}}$ or $S_{pO_{2}}$, respectively) is illustrated by the sigmoid‐shaped oxyhaemoglobin dissociation curve. Consequently, the HVR can be calculated using the formula: HVR=ΔV˙E/ΔV˙EΔSaO2ΔSaO2 (or $S_{pO_{2}}$). The HVR is usually determined in isocapnic conditions, meaning that $P_{\mathrm{aC}O_{2}}$ is held constant. However, if $P_{\mathrm{aC}O_{2}}$ is allowed to fall during hyperventilation (poikilocapnia), the HVR is lower. The significant individual variability in HVR (≤5‐fold difference) might help to explain susceptibility to or protection against hypoxia‐related pathologies (Oeung et al., 2023), such as AMS, HAPE, high‐altitude cerebral oedema and chronic mountain sickness (Gatterer et al., 2024). Although the HVR appears not to differ much between sexes (Tarbichi et al., 2003), factors such as genetics, ageing, certain diseases and endurance training can modify the HVR considerably (Janssen Daalen et al., 2024; Peterson et al., 1981; Scoggin et al., 1978; Weil, 2003). Moreover, the HVR is higher at high altitudes than at sea level and increases with the duration spent there (Oeung et al., 2023). The usual increase in HVR during acclimatization can be achieved in advance through pre‐acclimatization at terrestrial altitude and/or by breathing hypoxic air via face mask or spending time in hypoxic rooms or tents (J. Burtscher et al., 2026; Hupperets et al., 2004).

Ventilatory acclimatization depends on other factors in addition to the HVR, including changes in $P_{\mathrm{aC}O_{2}}$, HCO_3_^−^ and blood pH. Hyperventilation‐related respiratory alkalosis reduces the response of central chemoreceptors located on the ventral medullary surface of the brainstem and, consequently, the full response to hypoxia. Once ventilatory acclimatization is complete (usually after ∼1 week at high altitude), increased ${\dot{V}}_{E}$ and a related increase in $P_{aO_{2}}$ and $S_{pO_{2}}$ become possible despite the lower $P_{\mathrm{aC}O_{2}}$ values and alkalosis. Therefore, in addition to HVR and $S_{pO_{2}}$, information on changes in $P_{\mathrm{aC}O_{2}}$ and HCO_3_^−^ can help in assessing the status of ventilatory acclimatization.

## 4 EFFECTS OF DIFFERENT HYPOXIA EXPOSURES ON THE VENTILATORY RESPONSE TO HYPOXIA

### 4.1 Continuous exposure

Most available studies suggest a rapid increase in HVR during acclimatization to high altitude over 1 to 2 weeks at elevations between 4000 and 4500 m (Bender et al., 1989; Hupperets et al., 2004; Lenfant & Sullivan, 1971; White et al., 1987). For example, Sato et al. (1994) demonstrated that exposure of six healthy men to a terrestrial altitude of 3810 m for 12 days increased their isocapnic HVR by >90% (from 1.13 to 2.17 L/min/% $S_{aO_{2}}$). However, HVR did not plateau until day 12, and it returned to baseline after 4 days at sea level (Sato et al., 1994). During an 8‐week sojourn of four men and one woman at 3800 m, the isocapnic HVR increased by 130% within the first 24 h of exposure and remained stable during the first week (Hupperets et al., 2004). It then increased by a further 60% to the end of week 2, reaching its peak. After this, HVR decreased to values slightly above those seen at week 1 and remained stable until week 8 (Hupperets et al., 2004). A similar increase in HVR (136%) was observed in 15 young men and women during the first 24 h at an altitude of 3800 m, with a similar increase in both sexes (Basaran et al., 2016). Notably, this increase was significantly reduced by ibuprofen pretreatment. The authors speculated that ibuprofen might have affected ventilatory acclimatization via effects of nuclear factor‐κB on both cytokines and hypoxia‐inducible factor‐1α (Basaran et al., 2016). With regard to absolute altitude or the severity of hypoxia, Bhaumik et al. (2003) demonstrated that the HVR of eight young men and eight young women increased by almost 60% after ∼1 week of acclimatization at 2100 m when they ascended to 4350 m. Four to five days after descending to 2100 m, the HVR returned to baseline values. Kriemler et al. (2016) exposed 20 healthy prepubertal children aged 9–12 years, along with their fathers, to an altitude of 3450 m for 3 days. They demonstrated an increase in isocapnic HVR (adjusted for vital capacity) in adults from 0.174 L/L/% at low altitude to 0.208 and 0.261 L/L/% on days 1 and 3 at high altitude, and in children from 0.270 L/L/% to 0.277 and 0.358 L/L/%. Although the HVR was higher in children at low altitude, the increase at high altitude was similar for both children and adults. Changes in HVR from low to high altitude were significantly correlated (*r* = 0.61, *P* < 0.001) among child–father pairs, suggesting a partly hereditary influence (Kriemler et al., 2016).

A cautious summary of these results suggests that the HVR might more than double in comparison to sea level during a continuous stay at ∼4000 m. Most of the increase occurs within the first 24 h, with a further increase of approximately one‐third over the subsequent 2 weeks. Thereafter, the HVR appears to stabilize or decrease slightly over at least the following 6 weeks. It should be noted that, after months to years at high altitude, sensitivity to hypoxia can gradually decrease, attenuating the HVR and thereby reducing ventilation (West, 2006).

Although there can be significant differences in baseline levels (partly determined by genetics) (Kriemler et al., 2016; Oeung et al., 2023), there does not appear to be much difference in HVR changes in response to high altitude exposure between sexes or between children and adults. However, more studies directly comparing different age groups across both sexes are necessary. Notably, although the HVR and resting $S_{aO_{2}}$ values seem to increase in parallel, the $S_{aO_{2}}$ during exercise can improve further (by ≥3 weeks at altitude), despite the HVR plateauing earlier (Bender et al., 1989; Dunnwald et al., 2021). Given that diffusing capacity increases over time at altitude, $S_{aO_{2}}$ can improve even in the absence of further increases in HVR (Agostoni et al., 2011).

### 4.2 Intermittent exposure

Ventilatory long‐term facilitation (vLTF) and progressive augmentation can be initiated with hypoxic episodes as short as 40 s and with as few as three episodes (Mateika & Narwani, 2009; Mitchell et al., 2001; Panza et al., 2023). Enhancement of the HVR can persist for several days after acute IH (Foster et al., 2005), and vLTF is characterized by a progressive increase in respiratory motor output during normoxia following hypoxia exposure, which may last ≤90 min (Yokhana et al., 2011). vLTF is a consequence of the facilitation of the hypoglossal nerve, the genioglossus muscle and phrenic motoneurons, resulting in increased ${\dot{V}}_{E}$ (Yokhana et al., 2011). In humans, IH leads to vLTF only if CO_2_ is maintained at or above baseline levels (Puri et al., 2021). Sleeping in normobaric hypoxia leads to periodic breathing and elevated CO_2_ levels, particularly when using hypoxic tents (Abril et al., 2026; Pramsohler et al., 2019). Notably, changes in HVR and vLTF are closely related (Panza et al., 2023). It is therefore not surprising that different protocols of IH, in different conditions (mask breathing, room, tent, awake or asleep) can impact HVR and vLTF, hence ${\dot{V}}_{E}$ and $S_{pO_{2}}$. The results of studies using different exposure protocols over varying time periods are presented below in order to assess their possible effects on the (isocapnic) HVR; study characteristics are shown in Table 1.

**TABLE 1:** Study characteristics for (isocapnic) evaluation of the hypoxic ventilatory response after intermittent hypoxia exposures (sorted by increasing duration of daily intermittent hypoxia exposure).

Reference	Participants Age, years [mean (SD)]	IH design	$F_{IO_{2}}$ [$S_{aO_{2}}$]	Days (nights)	HVR base–peak; (day) (L/min/%$S_{aO_{2}}$), means
Foster et al. (2005)	9 men 25.7 (4.3)^a^	6 × 5 min/day	0.120	12	0.84–1.25; +49% (day 12)
Foster et al. (2005)	9 men 25.7 (4.3)^a^	30 min/day	0.120	12	0.84–1.15; +37% (day 12)
Koehle et al. (2007)	10 men 26.0 (2.1)^a^	12 × 5 min/day	0.120	7	0.47–0.75; +60% (day 4)
Koehle et al. (2007)	10 men 26.0 (2.1)^a^	1 h/day	0.120	7	0.47–0.79; +68% (day 7)
Katayama et al. (2009)	6 men 20.8 (2.2)	1 h/day	0.123	7	0.35–0.60; +71% (day 7)^b^
Wille et al. (2012)	13 men 24.9 (4.5)	1 h/day	0.126	7	0.73–1.10; +51% (day 7)
Lusina et al. (2006)	11 men 25.0 (1.0)	1 h/day	[80%]	10	0.30–0.61; +103% (day 10)
Garcia et al. (2000)	13 men 24.9 (4.5)	2 h/day	0.130	12	0.28–0.80; +186% (day 5)
Katayama et al. (2009)	6 men 20.8 (2.2)	3 h/day	0.123	7	0.38–0.58; +53% (day 7)^b^
Hoshikawa et al. (2015)	7 men 23.8 (3.0)	11–13 h/day (night)	0.164	(5)	0.26–0.75; +188% (day 5)

Abbreviations:$F_{IO_{2}}$, inspiratory fraction of oxygen; HVR: hypoxic ventilatory response; IH, intermittent hypoxia; $S_{aO_{2}}$, arterial oxygen saturation.

^a^ Data were reported for the overall sample.

^b^ Data have been estimated from a figure.

These normobaric IH studies (Table 1) demonstrated an increase in (isocapnic) HVR of between 43% and 188%. Those comparing the effects of repeated short‐term and long‐term IH exposure for 30–60 min/day over 7–12 days found no differences between the protocols (Foster et al., 2005; Koehle et al., 2007). Furthermore, it seems that, rather than hypoxia severity, the total duration of hypoxia exposure is more important for increasing HVR, provided a $P_{aO_{2}}$ threshold of ∼60 mmHg has been crossed. In this regard, Serebrovskaya et al. (1999) reported that mild hypoxia (end‐tidal $P_{O_{2}}$ of 70–80 mmHg) did not increase HVR, whereas severe hypoxia (end‐tidal $P_{O_{2}}$ of 45 mmHg) was followed by a 96% increase after brief daily IH exposures over 14 days. Support for the effects of pre‐acclimatization also comes from earlier studies, which demonstrated that combining continuous exposure to high altitude with hypobaric IH sessions resulted in elevated $S_{aO_{2}}$ values at rest and during exercise (Richalet et al., 1992; Savourey et al., 1994). Furthermore, intermittent exposure to high altitude among shift workers was shown to have a carryover effect on acclimatization lasting ≥5 days after returning to sea level (Brown, 1989). Collectively, these studies demonstrate that repeated IH consistently enhances the HVR, although the magnitude and time course vary with the exposure protocol. Daily hypoxic exposures of 30–60 min generally increase HVR by ∼40%–70%, whereas longer or more intensive protocols can produce increases approaching 100% or more. HVR typically reaches a peak within 4–12 days, can plateau or decline slightly with continued exposure, and often remains elevated for several days (e.g., 5–7 days) after the IH intervention before returning towards baseline. These findings indicate that relatively short, repeated hypoxic exposures are sufficient to induce substantial ventilatory acclimatization. Furthermore, they indicate that the effects are lost only a few days after the IH intervention (de‐acclimatization), which is important for the design of pre‐acclimatization protocols.

We can cautiously summarize that an increase in HVR of 50%–100% occurs after only a few short IH sessions (1 h/day for 7 days). Individuals with low baseline HVR are therefore likely to benefit from such short‐term exposures (like Case 1).

#### 4.2.1 Daytime and sleep dependence

The transition from wakefulness to sleep is characterized by a depression of respiratory chemoreflexes owing to increased thresholds and decreased sensitivity to CO_2_ (Stephenson et al., 2000). Individuals with sleep apnoea exhibited higher HVR and lower vLTF levels in the morning compared with the evening. This shift was amplified by daily exposure to IH (Gerst et al., 2011). Notably, HVR during sleep was reduced in young, healthy men. The decrease in HVR differed across sleep stages, significantly declining from 1.07 in wakefulness to 0.64 and 0.62 in stages 2 and 3/4, and to 0.33 L/min/%$S_{aO_{2}}$ during rapid eye movement sleep (Gerst et al., 2011; Oliveira et al., 2024). Taken together, these findings do not allow a clear conclusion to be drawn regarding the effect of repeated hypoxic sleep, as commonly practised by mountaineers preparing for high‐altitude climbs, on the course of ventilatory acclimatization.

#### 4.2.2 Effects of exercise

Following repeated training at simulated altitude, the HVR increased, whereas it did not after training at low altitude (Levine et al., 1992). The HVR is also known to increase during acute exercise (Pandit & Robbins, 1994). Rather than being the result of an interaction between the carotid chemoreflex and central command, this might be attributable to the synergistic interaction between the carotid chemoreflex and muscle mechano‐ and metaboreflexes (Oliveira et al., 2024). Support for this assertion comes from observations in patients with heart failure with reduced ejection fraction, in which interactions between the carotid chemoreflex and the muscle metaboreflex were demonstrated (Machado et al., 2020). This potentiation is prominent in healthy young humans during a variety of physical exertions, encompassing static and dynamic contractions of small and large muscle groups, at submaximal and maximal intensities (Oliveira et al., 2024). Evidence of hypoxic ventilatory decline in the first 15 min of exercise was shown when moderate‐intensity exercise was imposed on acute, sustained isocapnic hypoxia in close temporal proximity (Berdeklis et al., 2025). However, the ventilatory decline during exercise was less than the hypoxic ventilatory decline at rest, suggesting that exercise makes the ventilatory decline less steep (Berdeklis et al., 2025). Similar to repeated hypoxic sleep, the effect of exercise in hypoxia on ventilatory acclimatization remains unclear. However, our practical experience with pre‐acclimatization using a hypoxic tent suggests that both interventions might benefit ventilatory acclimatization when applied progressively over several weeks.

## 5 EFFECTS OF DIFFERENT HYPOXIC EXPOSURES ON RENAL COMPENSATION IN VENTILATORY ACCLIMATIZATION

### 5.1 Continuous exposure

Several studies have examined the time course of ventilatory and renal acclimatization during an extended stay at high altitude. There is general agreement that ventilatory and renal acclimatization are mostly completed after 5–10 days at altitudes between 4000 and 5000 m (Bender et al., 1989; M. Burtscher et al., 2025; Zouboules et al., 2018). This process varies with altitude, ranging from ∼4 days at 3000 m to between 4 and 6 weeks at altitudes of >6000 m (Hoiland et al., 2018). However, one previous study demonstrated that rapid renal compensation can occur within 24 h in healthy participants after acute ascent to 3800 m (Bird et al., 2021). On days 2 and 9 of this study, $P_{\mathrm{aC}O_{2}}$ and HCO_3_^−^ were lower, while arterial pH remained unchanged owing to renal compensation; renal reactivity did not increase between days 2 and 9 (Bird et al., 2021). We recently found that hyperventilation and increased diuresis occurred immediately upon exposure of healthy men and women to high altitude (3100 m), but renal compensation did not begin until 24 h (Skalla et al., 2026; Swenson et al., 1995). The sole metabolic response within the initial 24 h period was a slight decrease in plasma HCO_3_^−^. However, after 44 h had elapsed, more pronounced reductions in HCO_3_^−^ were observed, accompanied by a shift in trend in both altitude‐corrected base excess and arterial pH (Skalla et al., 2026). A previous study found that, in healthy men, ventilatory and renal acclimatization took only 2 days when ascending gradually, but 5 days when ascending rapidly by aircraft (Purkayastha et al., 1995). Thus, ventilatory and renal acclimatization to altitudes between 3000 and 4000 m can occur within 5 days, depending on the type of ascent. Figure 3 represents a schematic time course of ventilatory and renal acclimatization to those altitudes (Bird et al., 2021; Purkayastha et al., 1995; Skalla et al., 2026).

**FIGURE 3:**
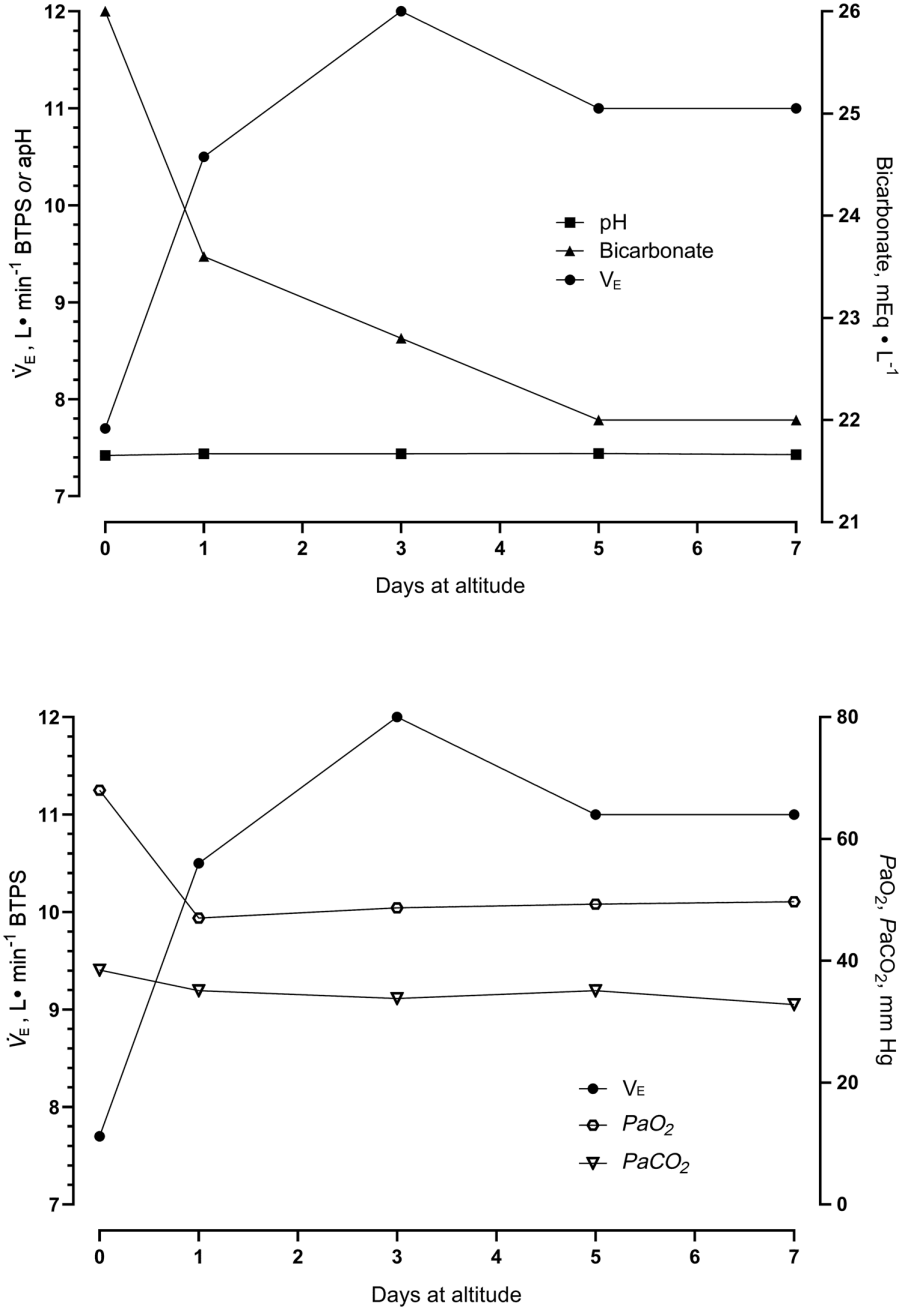
Schematic time course of ventilatory and renal acclimatization at altitudes between 3000 and 4000 m. The limited apparent variation in pH (top panel, squares) is attributable to the scaling of the *y*‐axis. The low resting ${\dot{V}}_{E}$ is attributable to the low body mass of the study participants. Abbreviations: $P_{\mathrm{aC}O_{2}}$, arterial partial pressure of carbon dioxide; $P_{aO_{2}}$, arterial partial pressure of oxygen; pH, arterial pH; ${\dot{V}}_{E}$, minute ventilation. Based on data from Bird et al. (2021), Purkayastha et al. (1995) and Skalla et al. (2026).

### 5.2 Intermittent exposure

The effects of IH on the HVR are described in detail above. This section summarizes the findings of various IH protocols involving different durations and severities of hypoxia, in addition to different indicators of ventilatory acclimatization, such as ${\dot{V}}_{E}$, $S_{pO_{2}}$ and blood gases. The existing literature is very limited, because only a few of these variables have been determined, and in different conditions (see Appendix Table A1). Overall, available evidence suggests that prolonged and repeated IH exposures (particularly protocols lasting several weeks, with several hours of daily exposure) are more likely to induce ventilatory and renal acclimatization, reflected by increased ventilation, improved oxygenation, reduced CO_2_ levels and enhanced renal compensation. However, shorter or less intense protocols show inconsistent effects, and the optimal exposure dose remains unclear. Moreover, the type of simulated altitude (normobaric vs. hypobaric hypoxia) has been reported to influence acclimatization processes, with hypobaric hypoxia being more effective in preventing AMS than normobaric hypoxia (Fulco et al., 2013). Furthermore, AMS symptoms can differ during the initial hours of altitude exposure between normobaric and hypobaric hypoxia (DiPasquale et al., 2016). Overall, interpretation is limited by heterogeneous study designs, small sample sizes and predominantly young male participants. Therefore, systematic studies are needed to define effective and individualized intermittent hypoxia protocols for pre‐acclimatization.

## 6 CURRENT KNOWLEDGE GAPS AND FUTURE RESEARCH DIRECTIONS

Although many of the key knowledge gaps were identified more than two decades ago (Powell & Garcia, 2000), the physiological mechanisms underlying IH, and how they differ from those induced by acute or chronic continuous hypoxia, remain incompletely understood. Although IH can enhance the HVR after only a few exposures, its effects on renal compensation and sustained ventilatory acclimatization remain poorly characterized. Consequently, current recommendations for IH‐based pre‐acclimatization rely largely on empirical experience rather than robust scientific evidence. Available data suggest that individuals with a low HVR might benefit from short IH protocols, whereas effective pre‐acclimatization before rapid ascent to high altitude is likely to require substantially longer exposures.

Future research should establish the optimal duration, intensity, timing and modality of IH (e.g., sleep vs. wakefulness, passive exposure vs. exercise, normobaric vs. hypobaric hypoxia) required to induce effective pre‐acclimatization. The relative contributions of ventilatory and renal adaptations, compared with haematological, metabolic, hormonal and cellular responses, also remain to be clarified. Well‐designed, long‐term randomized studies are needed to identify the most effective IH strategies across different populations, accounting for age, sex and individual susceptibility to altitude illness. Finally, studies evaluating combinations of IH with pharmacological interventions, such as carbonic anhydrase inhibitors, or with intermittent oxygen supplementation might determine whether pre‐acclimatization can be optimized while reducing the duration of IH exposure required.

## 7 CONCLUSION

The aim of this review is to summarize and critically evaluate the evidence on the potential of IH interventions to induce ventilatory acclimatization prior to high‐altitude exposure. Although it is clear that HVR can be increased by a few short hypoxic sessions, the available data on IH effects on renal compensation allow for only vague conclusions. Individuals with a generally reduced HVR, who might be potentially be susceptible to HAPE (Case 1) (M. Burtscher et al., 2008; Hackett et al., 1988), will benefit from a few IH sessions. For this purpose, for example, 1 h IH sessions (short cycles or continuous sessions) over 7 days are sufficient. Recommendations for effective pre‐acclimatization through IH exposure before a trekking tour or expedition are currently based primarily on experience, and the available studies cannot make them more evidence based. It certainly seems that longer periods of IH are necessary to achieve effective ventilatory and renal acclimatization, evidence supported by the reduction in AMS with increasing total hours of IH (ideally ≤200 h) (J. Burtscher et al., 2026). This is consistent with Case 2, which formed the basis of the pre‐acclimatization programme for rapid ascents to very high altitudes (M. Burtscher, 2025; Martin Burtscher et al., 2022). However, why several weeks of IH exposure are necessary to achieve favourable oxygen saturation and good tolerance at very high altitudes warrants further investigation. Given that a rapid ascent to altitudes of >4000 m requires several days of continuous exposure to allow for ventilatory acclimatization, it seems reasonable to assume that multi‐week IH exposures (e.g., sleeping in tents) involving progressive increases in simulated altitude are necessary to achieve similar adaptations. However, the question arises of whether it would be more comfortable to spend a few days at a natural high altitude than to sleep in a hypoxic tent for weeks. Whether ventilatory and renal acclimatization through IH are of primary importance or whether and to what extent haematological, metabolic, hormonal and other effects also play important roles remains to be clarified. If one decides to use IH for pre‐acclimatization, sleeping in a hypoxic tent appears to be the most feasible option. The inclusion of several exercise sessions in this environment should help to increase exercise tolerance in acute hypoxia (Katayama et al., 2001; Ricart et al., 2000). Nevertheless, systematic long‐term investigations of various IH strategies are necessary to clarify definitively which strategies are most suitable. It seems reasonable to evaluate the effects of IH with and without acetazolamide to determine whether IH could be replaced by carbonic anhydrase inhibitors (e.g., acetazolamide) or whether its duration could be significantly reduced.

## AUTHOR CONTRIBUTIONS

Conceptualization and first draft: Martin Burtscher. Writing—review & editing: Axel Kleinsasser, Katharina Hüfner, Johannes Burtscher, Erik Swenson, Peter Hackett and Hannes Gatterer. Tables and figures: Hannes Gatterer, Axel Kleinsasser and Martin Burtscher. All authors approved the final version of the manuscript and agree to be accountable for all aspects of the work in ensuring that questions related to the accuracy or integrity of any part of the work are appropriately investigated and resolved. All persons designated as authors qualify for authorship, and all those who qualify for authorship are listed.

## CONFLICT OF INTEREST

None declared.

## FUNDING INFORMATION

None.

## GENERATIVE AI STATEMENT

No artificial intelligence was used for the writing of this article. Figures 1 and 2 are our original figures, which have been slightly modified by the use of AI.

## 1

**TABLE A1:** Study characteristics on parameters of ventilatory acclimatization following various protocols of intermittent hypoxia exposures (sorted by the total amount of intermittent hypoxia duration).

Reference	Participants, Age	IH design	$F_{IO_{2}}$ PB or simulated altitude	Duration	${\dot{V}}_{E}$ (L/min)	$S_{pO_{2}}$ (%)	$P_{aO_{2}}$ (mmHg)	$P_{\mathrm{aC}O_{2}}$ ($P_{\mathrm{ETC}O_{2}}$) (mmHg)	HCO_3_^−^ (mmol/L)	pH	Base excess	Comments
					Changes/differences (before to after IH at altitude, or IH vs. CG)							
Gotschke et al. (2018)	IH: *n* = 8 (4 women) CG: *n* = 8 (4 women) 34 years	8 h/day, 5 days/week	2650 m HH	2.5–20 years (median 3.7 years)	—	*at 2650 m* IH: 93.1 ± 1.0 CG: 91.4 ± 2.5 *at SL* IH: 97.3 ± 0.4 CG: 97.0 ± 0.7	*at 2650 m* IH: 70.1 ± 7.3 CG: 63.2 ± 7.6 *at SL* IH: 86.4 ± 4.6 CG: 85.5 ± 5.9	*at 2650 m* IH: 28.5 ± 2.3 CG: 31.8 ± 2.4* *at SL* IH: 33.6 ± 4.6 CG: 37.7 ± 3.2*	—	*at 2650m* IH: 7.44 ± 0.01 CG: 7.44 ± 0.01 *at SL* IH: 7.43 ± 0.03 CG: 7.43 ± 0.02	*at 2650m* IH: −3.6 ± 1.1 CG: −2.3 ± 1.3* *at SL* IH: −0.9 ± 2.2 CG: 0.6 ± 1.1*	IH = workers on the Zugspitze (2650 m) Measurements were performed at 530 m and ∼2 h after passive arrival at 2650 m
Dehnert et al. (2014)	IH: *n* = 21 CG: *n* = 21 18–50 years	8 h/day	14%–15% (from 2500 m up to 3300 m) NH	14 nights	IH: 4.9 to 5.4 ^a^ CG: 5.3 to 5.2	IH: 78.5 to 81.2^a^ CG: 79.6 to 78.6	IH: 41.3 to 43.8^a^ CG: 42.5 to 42.5	IH: 34.7 to 34.8 CG: 34.2 to 34.9	—	—	—	Measurements were taken after a 4 h exposure to 12% $F_{IO_{2}}$ (4500m); ranges are given in the original article (no SD)
Hoshikawa et al. (2015)	*n* = 7 men 23 ± 3 years	11–13 h/day	16.4% (2000 m) NH	5 nights	—	93.8 ± 1.4 to 94.2 ± 1.1	—	41.5 ± 2.8 to 39.3 ± 3.5 measured in normoxia	—	—	—	Measurements during nights 1 and 5 (first vs. last)
Beidleman et al. (2004)	*n* = 5 men, *n* = 1 woman 23 ± 2 years	4 h/day, 5 days/week (*n* = 3 passive and *n* = 3 exercise first 60 min)	446 mmHg) 4300 m HH	3 weeks	—	80 ± 2 to 85 ± 1*	—	31.7 ± 0.7 to 28.0 ± 0.3*	—	—	—	30 h HA (4300 m) exposure Measurements taken after 20–22 h HA exposure Exercise did not affect any variable
Savourey et al. (1996)	*n* = 9 men 33 years old (SEM 2.3 years)	8 h/day	From 4500 to 8500 m (gradual increase) HH	5 days	12.7 (SEM 0.1) to 15.1 (SEM 0.1)*	88.8 (SEM 2.3) to 93.3 (SEM 1.1)*	54.6 SEM 2.7 to 53.2 SEM 1.4	26.1 SEM 1.6 to 21.8 SEM 0.9*	—	—	—	Measurements taken at 4500 m (PB = 589 hPa) (before vs. after)
Ricart et al. (2000)	*n* = 5 men, *n* = 4 women 44.7 years	2 h/day	5000 m HH	14 days	Resting: 17.3 ± 7.8 to 16.5 ± 6.8 Exercise (30%${\dot{V}}_{O_{2}\max}$ estimate): 55.5 ± 12.5 to 67.5 ± 22.5*	Exercise (30%${\dot{V}}_{O_{2}\max}$ estimate): 65.2 ± 9.4 to 71.5 ± 7.3*	—	—	—	—	—	Measurements taken at 5000 m
Beidleman et al. (2008)	*n* = 8 men, *n* = 2 women 26 ± 2 years	4 h/day, 5 days/week (*n* = 5 passive and *n* = 5 exercise, 45 min 70% HR_max_)	446 mmHg ∼4300 m HH	7 sessions within 9 days	—	Resting: 83 ± 2 to 86 ± 1* Exercise: 70% HR_max_: 75 ± 1 to 79 ± 1*	—	—	—	—	—	Measurements taken pre and post at 4300 m (PB 446 mmHg) 16% improvement in TT at 4300 m
Levine et al. (1992)	IH: *n* = 14 CG: *n* = 7 26–27 ± 3 years	45 min/day, 5 days/week	560 mmHg) ∼2500 m HH	5 weeks	—	2500 m IH: 92.3 ± 0.6 to 92.9 ± 0.3 CG: 91.1 ± 1.3 to 92.9 ± 0.3	$F_{IO_{2}}$: 12% IH: 48.5 ± 2.3 to 53.6 ± 2.1* $F_{IO_{2}}$: 8% IH: 32.0 ± 1.3 to 35.3 ± 1.2* CG: no changes	$F_{IO_{2}}$: 12% IH: 35.3 ± 0.8 to 34.2 ± 1.0 CG: no changes $F_{IO_{2}}$: 8% IH: 31.2 ± 0.8 to 30.2 ± 0.8 CG: 29.2 ± 1.2 to 33.0 ± 1.0*	—	$F_{IO_{2}}$: 12% IH: 7.45 ± 0.01 to 7.44 ± 0.01 $F_{IO_{2}}$: 8% IH: 7.49 ± 0.01 to 7.49 ± 0.01 CG: no changes	—	Blood gases assessed during the HVR test IH: ex training in hypoxia $S_{pO_{2}}$ at 2500 m
Lusina et al. (2006)	*n* = 11 men 25 ± 1 years old	1 h/day	$S_{pO_{2}}$ = 80% NH	10 days	9.8 ± 1.4 to 8.9 ± 1.6	80.4 ± 1.0 vs. 79.9 ± 0.3 $S_{pO_{2}}$ clamped to 80%	—	(41.3 ± 1.2 vs. 41.3 ± 1.7) isocapnic	—	—	—	Measurements performed after 20 min isocapnic hypoxia $S_{pO_{2}}$ 80% (pre vs. post IH)
Katayama et al. (2005)	*n* = 8 men 21.1 ± 1.5 years	1 h/day	12% NH	2 × 10 days separated by 1 month	Series 1: ∼10.3 ± 0.9 to 11.6 ± 5 Series 2: 11.6 ± 2.2 to 9.5 ± 2.3	Series 1: 70 ± 7 to 77 ± 9* Series 2: 75 ± 8 to 75 ± 7	—	Series 1: 40 ± 1 to 38 ± 4 Series 2: 38 ± 3 to 38 ± 2	—	—	—	Measurements taken during the last 20 min of the 1 h sessions First vs. last session
Katayama et al. (2001)	*n* = 6 men 21.5 ± 3.6 years old	1 h/day + 30 min decompression	432 mmHg ∼4500 m HH	7 days	Resting: 10.4 ± 2.1 to 12.8 ± 2.0* Exercise (70%${\dot{V}}_{O_{2}\max}$): 84.8 ± 18.2 to 94.8 ± 17.1*	Resting: ∼67 ± 6 to 72 ± 6* Exercise: (70%${\dot{V}}_{O_{2}\max}$): ∼60 ± 6 to 67 ± 5*	—	—	—	—	—	Measurements performed at 4500m (432 mmHg) No changes in *W*_max_ and ${\dot{V}}_{O_{2}\max}$
Wille et al. (2012)	*n* = 26 men 26 ± 5 years old IH: *n* = 13 CG: *n* = 13	1 h/day	12.6% ∼4500 m NH	7 days	—	IH: 78.9 ± 4.8 to 80.5 ± 3.5 CG: 96.5 ± 0.5 to 96.7 ± 0.9	—	—	—	—	—	8 h at $F_{IO_{2}}$ 11.3 (∼5300 m) $S_{pO_{2}}$ first vs. last session (values in table) IH and CG: no differences in $S_{pO_{2}}$ during the 8 h HA stay
Faulhaber et al. (2016)	*n* = 32 IH: 17 (8 men) CG: 15 (7 men) 38 ± 11 years	1 h/day	12.6% ∼4500 m NH	7 days	IH vs. CG: 21 h: 11.7 ± 2.8 vs. 10.0 ± 2.5	IH vs. CG: 6 h: 85.8 ± 3.0 vs. 85.2 ± 3.8 45 h: 88.1 ± 2.4 vs. 87.4 ± 2.6	IH vs. CG: 6 h: 49.9 ± 3.9 vs. 48.9 ± 4.7 45 h: 54.5 ± 3.6 vs. 52.9 ± 3.6	IH vs. CG: 6 h: 30.0 ± 3.1 vs. 31.1 ± 4.4 45 h: 27.1 ± 2.1 vs. 28.4 ± 2.3	IH vs. CG: 6 h: 21.2 ± 1.8 vs. 21.6 ± 2.8 45 h: 18.8 ± 1.9 vs. 19.2 ± 1.5	—	—	Blood gas measurements taken after 6 and 45 h at an altitude of 3650 m Ventilation after 21 h No effects on AMS prevention
Treml et al. (2020)	*n* = 49 IH: *n* = 23 (11 men) CG: *n* = 26 (14 men) 24 years	1 h/day	12.6% ∼4500 m) NH	7 days	—	IH: 81.2 to 84.5 CG: 81.5 to 83.3	IH: 36 to 39 CG: 41 to 38	IH: 32 to 33 CG: 33 to 34	—	IH: 7.45 to 7.46 CG: 7.46 to 7.46	—	12 h at 4500 m pre and post IH Blood gases after 6 h
Katayama et al. (2007)	*n* = 18 men ∼20 years IH1: *n* = 6 IH2: *n* = 6 CG: *n* = 6	1 h/day	15.5% (IH1) 12.3% (IH2) NH	7 days	Resting: IH1: 11.6 ± 2.3 to 12.0 ± 1.1 IH2: 12.4 ± 1.3 to 11.4 ± 1.4 Exercise 70% pre‐${\dot{V}}_{O_{2}\mathrm{peak}}$: IH1: 86.4 ± 18.4 to 84.0 ± 16.8 IH2: 78.3 ± 8.8 to 73.0 ± 7.7	No effect of IH (resting, 40% and 70% of pre‐${\dot{V}}_{O_{2}\mathrm{peak}}$ and ${\dot{V}}_{O_{2}\max}$)	—	—	—	—	—	Parameters pre vs. post IH at 2500 m ($F_{IO_{2}}$ 15.5) No change in exercise performance

Abbreviations: AMS, acute mountain sickness; CG, control group; $F_{IO_{2}}$, inspiratory fraction of oxygen; HCO_3_^−^, arterial bicarbonate; HH, hypobaric hypoxia; HR_max_, maximal heart rate; IH, intermittent hypoxia; NH, normobaric hypoxia; $P_{\mathrm{aC}O_{2}}$, arterial partial pressure of carbon dioxide; $P_{aO_{2}}$, arterial partial pressure of oxygen; PB, barometric pressure; $P_{\mathrm{ETC}O_{2}}$, end‐tidal partial pressure of carbon dioxide; $S_{aO_{2}}$, arterial oxygen saturation; $S_{pO_{2}}$, peripheral oxygen saturation; ${\dot{V}}_{E}$, minute ventilation; ${\dot{V}}_{O_{2}\max}$, maximal oxygen uptake; ${\dot{V}}_{O_{2}\mathrm{peak}}$, peak oxygen uptake; *W*_max_, maximal work rate.

^a^ Insignificant trend. *indicates the difference is statistically significant.
